# Association between person and disease related factors and the planned diabetes care in people who receive person-centered type 2 diabetes care: An implementation study

**DOI:** 10.1371/journal.pone.0219702

**Published:** 2019-07-24

**Authors:** Heidi A. van Vugt, Eelco J. P. de Koning, Guy E. H. M. Rutten

**Affiliations:** 1 Department of General Practice, Julius Center for Health Sciences and Primary Care, University Medical Center Utrecht, Utrecht University, Utrecht, the Netherlands; 2 Department of Medicine, Leiden University Medical Center, Leiden, the Netherlands; Chinese Academy of Medical Sciences and Peking Union Medical College, CHINA

## Abstract

**Aims:**

To assess the planned diabetes care for the coming year and its associated factors in patients with Type 2 diabetes who have a person-centered annual consultation.

**Methods:**

Implementation study of a new consultation model in 47 general practices (primary care) and 6 outpatient clinics (secondary care); 1200 patients from primary and 166 from secondary care participated. Data collection took place between November 2015 and February 2017. Outcomes: preferred monitoring frequency; referral to other health care provider(s); medication change. One measurement at the end of the consultation. We performed logistic regression analyses. Differences between primary and secondary care were analyzed.

**Results:**

Many patients arranged a monitoring frequency <4 times per year (general practices 19.5%, outpatient clinics 40%, p < .001). Type of provider (physician/nurse, OR 3.83, p < .001), baseline HbA1c (OR 1.02, p = .017), glucose lowering medication; and setting treatment goals (OR .65, p = .048) were associated with the chosen frequency. Independently associated with a referral were age (OR .99, p = .039), baseline glucose lowering medication and patients’ goal setting (OR 1.52, p = .016). Medication change was associated with type of provider, baseline HbA1c, blood glucose lowering medication, quality of life (OR .80, p = .037) and setting treatment goals (OR 2.64, p = .001).

**Conclusions:**

Not only disease but also person related factors, especially setting treatment goals, are independently associated with planned care use in person-centered diabetes care.

## Introduction

In 2007, the Dutch government introduced bundled payment to facilitate ‘disease management’ for people with diabetes with an annual lump sum for an individual’s diabetes care, medications not included. This triggered important improvements in the organization of diabetes care, especially with regard to the coordination and evidence-based nature of care [1,2].

In the Netherlands, about 85–90% of patients with Type 2 diabetes are treated by a general practitioner and/or a practice nurse in primary care (PC). Almost all general practices are organized within the framework of a care group that may consist of 15 to 200 general practices. Patients are referred to hospital outpatient clinics (secondary care (SC)) when they need more complex diabetes care. They are treated by an internal medicine specialist/endocrinologist and a diabetes specialized nurse [3].

According to the bundled payment agreement almost all patients are monitored four times a year. Both the European and American diabetes care organizations state that diabetes treatment should integrate patient’s preferences, needs, values and self-management possibilities [4]. Patient involvement in the current disease-management system is limited and self-management support strategies remain relatively underdeveloped in the Netherlands^3^ and in many other countries [5–8].

To facilitate person-centered care including shared decision making, the Dutch Diabetes Federation developed a four steps consultation model for the annual diabetes consultation. The model integrates patients’ preferences, needs, values and self-management possibilities. It is currently implemented in Dutch general practices and outpatient clinics [9]. Using the model, healthcare providers get informed not only about disease related factors such as glycemic control and comorbidities, but also about individual’s life related factors that influence diabetes self-management, such as illness perception, quality of life, diabetes distress and a patient’s social context. We could demonstrate that the model is well applicable [9]. People with Type 2 diabetes and care providers perceive shared decisions about treatment and care in almost all consultations [9]. To what extent healthcare utilization varies within a person-centered approach is unknown. Here we report 1) the type and amount of care people with Type 2 diabetes plan after the person-centered annual consultation with regard to the monitoring frequency, referrals to other healthcare provider(s) and the change in medication, and 2) what factors were associated with these outcomes in people with a more or a less complex type diabetes.

## Materials and methods

### Study design and setting

This is an implementation study with a measurement of the planned intended Type 2 diabetes care in the year after the use of a new diabetes consultation model. The consultation model was implemented in general practices and outpatient clinics in the Netherlands from November 2015 until September 2016 in a representative group of 47 general practices (57 general practitioners and 23 practice nurses) and six outpatient clinics (17 medical specialists and 8 diabetes specialist nurses) [9]. They invited patients ≥18 years with Type 1 or Type 2 DM, on the condition that people were capable of filling out questionnaires. Inclusion ended on February 1 2017. As described in detail previously [9], participants were sent an information letter about the new consultation or received the letter from their healthcare provider a month before their annual consultation. If they were willing to participate, they were recommended to prepare four questions: 1. Do you have health problems?; 2. Would you like to solve your health problems?; 3. How would you like to do that?; 4. What type of support do you need?

In total 2,617 people with Type 1 or Type 2 diabetes mellitus were invited; 1,487 participated [9]. Here we report about a representative sample of 1366 patient with Type 2 diabetes; 1200 (87.8%) being treated in PC and 166 (12.2%) in SC; 895 patients (65.5%) had a physician-led conversation (65.5%) and 471 (34.5%) a nurse-led conversation. [9]. No ethical approval was needed according to the Local Ethics Committee of the University Medical Center Utrecht, because the study did not fulfill the criteria for medical human scientific research under Dutch legislation. All study procedures were in accordance with the Declaration of Helsinki. All patients gave written informed consent.

### The consultation model

The four steps consultation model has been described in detail previously [9]. In the first step the diabetes care provider discusses disease and person related factors with the individual, such as diabetes related complications, cardiovascular risk, glycemic control and medication use on the one hand and person related factors such as lifestyle, motivation, self-management possibilities, illness perceptions, quality of life, and social aspects on the other. Care provider and patient discuss the topics that are actually relevant. In step 2–4 shared decisions are made regarding to personal goals, treatment, and the diabetes care needed for the upcoming year. Diabetes care providers were trained two times two hours to use the consultation model as described above during the annual diabetes consultation [9].

### Patient and public involvement

Individuals of the Dutch Diabetes Association (DVN) were involved in the development of the consultation model, the four questions to prepare the consultation and the questionnaires which patients filled out after the consultation.

### Planned utilization of diabetes care services

Utilization of diabetes care services for the year after the person-centered consultation consisted of 1. the preferred monitoring frequency with the general practice or outpatient clinic (<4, 4, >4 times a year); 2. Any referral to other healthcare providers such as other medical specialists, a dietician, physiotherapist, psychologist, podiatrist or lifestyle coach. 3. Medication change; the change in blood glucose, lipid and/or blood pressure lowering medication; in dosing and/or adding and/or ending a drug.

### Data collection and variables

#### Diabetes care providers’ questionnaire

Physicians and nurses were requested to complete an online questionnaire immediately after each consultation. The questionnaire contained questions about individual’s personal goals, the preferred number of monitoring visits during the forthcoming year, referral(s) to other healthcare providers and (change in) medication.

#### Patients’ questionnaire

Patients filled out a set of questionnaires before the consultation which contained:

- A study-specific questionnaire on sex, ethnicity, marital status, level of education, employment status, diabetes duration, alcohol use and comorbidities [9]. A statement on social support, namely ‘People around me support me when I have health-related problems’ that could be answered with: ‘strongly agree’ (a lot), ‘agree’ (moderate), disagree’ (a little) or ‘strongly disagree’ (hardly any).
- The EuroQol 5D (EQ-5D), a frequently used and validated health status questionnaire that consists of following five dimensions; mobility, self-care, daily activities, pain/discomfort, and anxiety/depression. These dimensions can be rated from ‘no problems’ to ‘severe problems’. The total score ranges from -0.33 to 1.00 with a lower score reflecting a worse health status [10].
- The Audit of Diabetes-Dependent Quality of Life (ADDQoL) that measures the impact of diabetes and its treatment on quality of life. It is validated and consists of 19 questions on which an average weighted impact is calculated that ranges from -9 to 3. A lower score indicating a more negative influence on quality of life [11].
- The validated Brief Illness Perception Questionnaires (BIPQ) that evaluates illness perception and consists of eight items: consequences, timeline, personal control, treatment control, identity, illness concern, coherence and emotional representation. These items are rated on a 0–10 scale [12]. A higher score reflects a stronger belief in their treatment and/or control, a greater perceived impact of aspects of T2DM, a better understanding of T2DM.
- The validated 5-item Problem Areas In Diabetes scale (PAID-5) that measures diabetes-related distress. The score on each item ranges from 0 (not a problem) to 4 (serious problem). A total score of ≥8 may indicate severe diabetes related distress [13].
- The validated Summary of Diabetes Self-Care Activities Measure (SDSCA) that to measures e diabetes self-management over the previous seven days in five domains; general diet, specific diet, physical exercise, blood glucose testing, foot care, and one additional question about smoking status [14,15].
- The Patient Activation Measure (PAM-13), a 13-item validated instrument that assesses an individual self-reported knowledge, skills, and confidence to engage in for self-management activities [9,16]. The mean PAM-13 score is transformed into a score ranging from 0 to 100; a with here higher scores refers to presenting higher activation.

We extracted data on diabetes type, age, glycated hemoglobin (HbA1c), lipids, blood pressure and Body Mass Index from patient’s electronic medical record. The HbA1c level is a marker of the average blood glucose levels over a period of the last 2–3 months.

### Statistical analysis

Continuous normally distributed data are summarized using means and standard deviations (SD) and continuous non-normally distributed data with medians and 25–75% interquartile ranges (IQR). Categorical data are reported as counts and percentages. Means were compared using Student’s t-test or Mann Whitney U test for unpaired samples and Chi square test was used for proportionate samples to assess differences between participants from PC and SC.

A multivariable binary logistic regression analysis was performed to assess the person and disease related factors which influence the preferred monitoring frequency after the consultation (4 or more vs. less than 4 times a year), referral to other healthcare professionals (yes vs. no) and change in medication (yes vs. no). A binary logistic regression analysis was also performed for “monitoring frequency”, because numbers in one of the three categories, namely ‘>4 times’ were too small. We controlled for patients’ characteristics that differed between PC an SC and included factors that were considered relevant from the literature, namely age, education level, marital status, ethnicity, comorbidity, illness duration, HbA1c, lipids, blood pressure, BMI, quality of life, mental distress, and medication use [17–22]. We also included the patient activity score, their social support, having treatment goals or not, and type of healthcare provider (physician vs. nurse) as potential confounders based on our clinical view.

We used multiple imputation for handling missing data in the multivariable analyses, because the exclusion of patients with missing values can lead to biased results. We report the estimates based on the pooled results of five imputed datasets.

Healthcare providers reported patients’ goals using open-ended items. These were grouped and counted by one author (HAvV) and an independent policy maker from the Dutch Diabetes Federation (CB). Disagreement was resolved in consensus.

Marital status was recoded into ‘married / cohabitating’ and ‘single’ (divorced, not or never married or widow). Educational level was recoded into ‘low’ (no education, primary school or lower education), ‘intermediate’ or ‘high’ (higher education or university degree). For the PAM, patients who filled out less than 10 items or who answered all items with ‘disagree strongly’ or ‘agree strongly’ were excluded. Mean scores were calculated leaving out items that were deemed not applicable by the respondents, and then transformed into a standardized activation score ranging from 0 to 100, based on a conversion table provided by the developers [16]. The two domain specific diet items from the Summary of Diabetes Self-Care Activities Measure are reported separately because of the low inter-item correlation [15].

Analyses were performed using SPSS version 23.0 (SPSS INC, Chicago, IL, USA). A p-value <0.05 was considered significant.

## Results

### Study population

Table 1 shows the characteristics of the study population, some of which have been reported previously [9]. Of all participants in PC 41.2% were female and 93.2% of Caucasian ethnicity. Their mean (sd) age was higher compared to patients in SC (66.1 (9.7) vs. 64.1 (10.1), p = .011), and they had a significantly shorter diabetes duration, a lower median BMI and a better mean HbA1c (9). The use of blood glucose lowering medication differed significantly between PC and SC with more than 90% of people treated in SC using insulin (p < .001). In SC also more patients used blood pressure lowering medication than in PC, 84.5 vs. 75.9% (p = .022). Self-care activities differed between participants treated in PC and SC with regard to blood glucose testing; 1.0 vs. 4.2 days per week (p < .001) and foot-care; 1.5 vs. 2.4 days per week (p < .001). People who were treated in PC had higher median (IQR) scores on the EQ-5D (0.84 (0.79–1.00) vs. 0.81 (0.72–1.00), p = .004) and on the ADDQol suggesting a better diabetes related quality of life than people treated in SC (-.29 (-1.00-.00) vs. -1.3 (-2.36—.61), p < .001). Also their diabetes distress level was significantly lower (median PAID-scores 3 (1–7) and 6 (3–10), p < .001). Patients in SC were not only more conscious about the consequence of their diabetes and its duration, they had a better understanding of the disease, but also more illness concern. Their diabetes had more impact (emotional representation) than in patients in PC. (p < .001). Most of all patients experienced a moderate social support (PC 67.9 and SC 70.0%).

**Table 1:** Patient characteristics. Means (±SD) or percentages, unless indicated otherwise*.

		PC (n = 1200)		SC (n = 166)			Total population (n = 1366)	
		n		n		p**	n	
Age (years)		1199	66.1 (9.7)	166	64.1 (10.1)	.011	1365	65.9 (9.8)
Female gender		1132	41.2	157	44.6	.437	1289	41.6
Ethnicity, Caucasian		1132	93.2	156	90.4	.243	1288	92.9
Marital status; married or cohabiting		1135	77.4	156	69.9	.044	1291	76.5
Educational level		1127		154		.437	1281	
*Low / Intermediate / High*			35.0/44.5/20.5		29.9/47.4/22.7			34.4/44.9/20.7
Employment status; having a paid job		1102	28.9	152	27.6	.775	1254	28.8
Smoking		1117	13.1	155	22.6	.003	1272	14.2
Alcohol use (yes)		1113	48.3	153	39.9	.057	1266	47.3
Illness duration (years, median, IQR)		1095	8 (4–14)	147	18 (12–25)	< .001	1242	10 (5–16)
Number of comorbid conditions (median, IQR)		1114	1 (1–2)	153	2 (1–3)	< .001	1267	1 (1–3)
HbA1C (% / mmol/mol)		1135	6.9 (0.9) /52 (9.5)	159	7.9 (1.2) /63 (13.3)	< .001	1294	7.0 (1.0) /53 (10.6)
SBP (mmHg)		1132	136.0 (14.9)	158	141.3 (19.9)	< .001	1290	136.7 (15.7)
LDL cholesterol (mmol/mol)		1127	2.4 (0.9)	137	2.4 (0.9)	.681	1264	2.4 (0.9)
BMI (kg/m^2^)(median, IQR)		1134	29.2 (26.3–32.9)	152	30.7 (27.8–34.9)	.001	1286	29.4 (26.4–33.1)
Baseline blood glucose-lowering medication		1140		148		< .001	1288	
*No medication*			21.5		0.7			19.1
*Oral medication only*			62.2		6.8			55.8
*Oral medication and insulin*			13.0		49.3			17.2
*Insulin only*			3.3		43.2			7.9
Baseline lipid lowering medication		1138	77.9	148	79.7	.673	1286	78.1
Baseline blood pressure lowering medication		1139	75.9	148	84.5	.022	1287	76.9
PAM		1042	59.1 (11.8)	146	58.3 (11.2)	.510	1188	58.9 (11.7)
SDSCA								
*General diet*		1047	4.7 (2.0)	152	4.8 (1.8)	.511	1199	4.7 (1.9)
*Specific diet*;	*Fruit*	1084	5.2 (2.0)	154	5.1 (2.0)	.582	1238	5.2 (2.0)
	*Fat*	1078	4.3 (2.2)	153	4.2 (2.1)	.297	1231	4.3 (2.1)
*Physical exercise*		1065	4.1 (2.0)	152	3.9 (2.1)	.276	1217	4.0 (2.0)
*Blood glucose testing*		1007	1.0 (1.9)	147	4.2 (2.5)	< .001	1154	1.4 (2.3)
*Foot-care*		1052	1.5 (2.0)	150	2.4 (2.2)	< .001	1202	1.6 (2.1)
EQ-5D (median)		1065	0.84 (0.79–1.00)	143	0.81 (0.72–1.00)	.004	1208	.84 (.78–1.00)
ADDQol (median, IQR)		1096	-.29 (-1.00 -.00)	150	-1.3 (-2.36–-.61)	< .001	1246	-.36 (-1.19—.06)
PAID (median, IQR)		1107	3 (1–7)	154	6 (3–10)	< .001	1261	3 (1–7)
BIPQ								
*Consequence*		1105	3.9 (2.6)	154	6.2 (2.1)	< .001	1259	4.1 (2.7)
*Timeline*		1077	8.4 (2.6)	152	9.5 (1.5)	< .001	1229	8.5 (2.4)
*Personal control*		1102	6.8 (2.2)	156	6.8 (2.0)	.824	1258	6.8 (2.2)
*Treatment control*		1093	7.4 (2.2)	152	7.7 (1.9)	.329	1245	7.5 (2.2)
*Identity*		1104	3.2 (2.5)	155	5.6 (2.5)	< .001	1259	3.5 (2.7)
*Illness concern*		1106	4.5 (3.0)	155	6.1 (2.6)	< .001	1261	4.7 (3.0)
*Coherence*		1093	6.9 (2.3)	155	7.3 (1.7)	.119	1248	7.0 (2.2)
*Emotional representation*		1102	2.8 (2.8)	155	5.1 (2.8)	< .001	1257	3.1 (2.9)
Social support		1088		150		.532	1238	
*A lot / Moderately / A little / Hardly*			25.0 / 67.9 / 5.2 / 1.8		21.3 / 70.0 / 8.0 / 0.7			24.6 / 68.2 / 5.6 / 1.6

PC: Primary Care; SC: Secondary care; HbA1c: glycosylated hemoglobin; SBP: systolic blood pressure; LDL: low-density lipoprotein; BMI: body mass index; PAM: Patient Activation Measure; EQ-5D: EuroQol 5D; SDSCA: Summary of Diabetes Self-Care Activities Measure; ADDQol: Audit of Diabetes Dependent Quality of lift; PAID: Problem Areas In Diabetes Scale; BIPQ: Brief Illness Perception Questionnaire

* some outcomes were reported earlier (Rutten, 2018; reference 9)

** Significant (p<0.05) difference between PC and SC

### Treatment goals, intended diabetes care services and medication change

Diabetes care providers reported 1502 personal treatment goals in 989 individuals. Patients had more often personal goals regarding lifestyle change in PC than in SC (31.7 vs.16.9%, p = < .001). In SC patients had more often goals regarding better cardiometabolic control (24.2 vs. 15.7%, p = .002), no or limiting hypoglycemia (7.4 vs. 0.9%, p < .001) and improving psychological well-being (8.7 vs. 3.6%, p = .001). They also put more emphasis on self-management (6.9 vs. 3.8%, p = .049) compared to patients in PC. (Table 2).

**Table 2:** Patient goals in primary (PC) and secondary care (SC) (n, %).

	PC (n = 1200)		SC (n = 166)			Total population (n = 1366)	
	n	%	n	%	p*	n	%
Patient who set goals together with diabetes care provider	859	75.7	130	89.0	< .001	989	77.2
Personal and disease related goals (more goals per patient possible)	1271	100	231	100		1502	100
*Lifestyle change*	403	31.7	39	16.9	< .001	442	29.4
*Quit or reduce smoking*	25	2.0	2	0.9	.415	27	1.8
*Better cardiometabolic control (blood glucose*, *lipids*, *blood pressure)*	200	15.7	56	24.2	.002	256	17.0
*No or limiting hypoglycemia*	11	0.9	17	7.4	< .001	28	1.9
*Loss of body weight*	229	18.0	30	13.0	.072	259	17.2
*Improve psychological well-being*	46	3.6	20	8.7	.001	66	4.4
*Prevent complication and/or reduce physical complaints*	92	7.2	17	7.4	.891	109	7.3
*No change in current policy (no specific goal)*	178	14.0	23	9.9	.114	201	13.4
*More diabetes education*	39	3.0	11	4.7	.228	50	3.3
*More emphasis on self-management*	48	3.8	16	6.9	.049	64	4.3

* Significant (p<0.05) difference between PC and SC

Table 3 shows the planned utilization of diabetes care services in the year after the person-centered annual review and change in medication during the annual review. The preferred number of monitoring visits with the general practice or outpatient clinic demonstrates the different care paths with 40% of patients treated by medical specialists and diabetes nurses arranging less than four visits. In SC shared decisions were more often made to consult other medical specialists (35.1 vs 11.7%, p < .001) and/or a dietician (30.4 vs. 17.9%, p = .001). During the consultation the healthcare provider changed medication; in PC 14.8% and in SC 27.7% (p < .001). Blood glucose lowering medication was most frequently changed.

**Table 3:** Preferred monitoring frequency, referral to other healthcare providers and medication change after the patient-centered consultation (n, %).

	PC (n = 1200)		SC (n = 166)			Total population (n = 1366)	
	n		n		p*	n	
Preferred monitoring frequency for the coming year*	1133		145		< .001	1278	
*>4 times*		4.5		13.1	< .001		5.5
*4 times*		76.0		46.9	< .001		72.7
*<4 times*		19.5		40.0	< .001		21.8
Referral to other healthcare providers in the coming year	1141		148		.353	1289	
Other doctors*		11.7		35.1	< .001		14.4
Dietician*		17.9		30.4	.001		19.3
Physiotherapist		6.1		4.7	.584		6.0
Psychologist		1.8		4.1	.115		2.1
Podiatrist		4.9		5.4	.840		5.0
Lifestyle coach		2.3		2.7	.770		2.3
Overall change in medication*	1136	14.8	148	27.7	< .001	1284	16.3
*Change in blood glucose lowering medication (number of times)*							
*Stopped / less*		79		23			102
*Started / more*		12		6			18
*Change in blood pressure lowering medication (number of times)*							
*Stopped / less*		2		1			3
*Started / more*		-		1			1
*Change in lipid lowering medication (number of times)*							
*Stopped / less*		8		-			8
*Started / more*		2		-			2

* Significant (p<0.05) difference between PC (primary care) and SC (secondary care)

### Factors determining monitoring frequency, referral to other healthcare providers and change in medication

Before imputation, 7.1% of all values were missing, distributed among 592 cases (43.3%). After controlling for possible confounders, patients who had their consultation with a physician more often planned a monitoring frequency of at least four times a year than patients who had their consultation with a nurse (OR 3.83, CI 2.64–5.59; p < .001). The same applied to patients who used oral blood glucose lowering medication compared to those patients who did not (OR 2.30, CI 1.47–3.60; p = < .001). A planned monitoring frequency of at least four times a year was also associated with a higher HbA1c (OR 1.02, CI 1.00–1.04; p = .017) and with patients who less often set treatment goals (OR .65, CI .43–1.00; p = .048).

Patients who were referred to other healthcare providers used more often a combination of oral blood glucose lowering medication and insulin (OR 2.67, CI 1.64–4.35; p < .001) or insulin only (OR 3.08, CI 1.47–6.47; p = .004), and/or blood pressure lowering medication (OR 1.39, CI 1.03–1.89; p = .033) and/or needed foot care (OR 1.06, CI 1.00–1.13; p = .048) and/or had set treatment goals (OR 1.52, CI 1.09–2.13; p = .016). Patients who were younger (OR .99, CI .97–1.00; p = .039) and/or those who used lipid lowering medication (OR .73, CI.55-.97; p = .028) were less often referred to other healthcare providers.

After controlling for possible confounders, patients with a higher HbA1c (OR 1.02, CI 1.00–1.03; p = .036), those who used blood glucose lowering medication and/or those with personal treatment goals (OR 2.64, CI 1.54–4.55; p = .001) were more likely to change medication. On the contrary, patients who had the consultation with a physician less often changed medication (OR .32, CI .21-.49; p < .001) compared to those who consulted a nurse. Patients with higher diabetes related quality of life had a lower chance of medication changes (OR .80, CI .65-.99; p = .037). (Table 4).

**Table 4:** Multivariable binary logistic regression analysis of factors associated with intended monitoring frequency, referral to other healthcare providers and change in medication use (total study population, n = 1366).

Patient characteristics	Monitoring frequency at least four / year			Referral to other healthcare providers			Change in medication		
	OR	95% CI OR	p-value	OR	95% CI OR	p-value	OR	95% CI OR	p-value
Physician (vs. nurse)	3.83	2.64–5.59	< .001*	.83	.65–1.07	.106	.32	.21-.49	< .001*
Age (years)	1.00	.99–1.02	.750	.99	.97–1.00	.039*	1.02	.99–1.04	.208
Caucasian (vs. non-Caucasian)	.75	.43–1.32	.315	1.15	.76–1.74	.513	1.62	.56–4.66	.343
Married/cohabitating (vs. singe)	.92	.65–1.29	.612	1.09	.83–1.44	.536	.89	.61–1.32	.568
Educational level—Low (ref)									
Intermediate educational level	.95	.67–1.34	.761	.77	.59–1.00	.053	1.10	.74–1.62	.641
High educational level	1.13	.72–1.77	.600	.85	.61–1.17	.313	1.12	.70–1.80	.642
Smoking (vs. non smoking)	.78	.50–1.22	.271	.75	.52–1.07	.112	1.28	.80–2.03	.302
Illness duration (years)	1.00	.98–1.02	.998	1.00	.99–1.02	.903	1.00	.97–1.02	.714
Number of comorbid conditions	1.07	.973–1.17	.167	1.02	.95–1.09	.598	.99	.90–1.09	.834
HbA1c (mmol/mol)	1.02	1.00–1.04	.017*	.99	.97–1.00	.116	1.02	1.00–1.03	.036*
Systolic blood pressure (mmHg)	.99	.98–1.00	.158	1.00	.99–1.01	.845	1.01	.99–1.02	.339
Body Mass Index (kg/m^2^)	1.00	.97–1.02	.721	.99	.97–1.02	.558	.97	.94–1.00	.083
Baseline blood glucose lowering medication (vs. no medication)									
*Oral medication only*	2.30	1.47–3.60	< .001*	.99	.70–1.41	.953	2.71	1.39–5.31	.004*
*Oral medication and insulin*	1.33	.71–2.513	.372	2.67	1.64–4.35	< .001*	3.15	1.36–7.29	.008*
*Insulin only*	.98	.47–2.06	.954	3.08	1.47–6.47	.004*	4.28	1.55–11.84	.006*
Baseline BP lowering medication (vs. no medication)	1.23	.86–1.76	.248	1.39	1.03–1.89	.033*	1.12	.73–1.71	.601
Baseline lipid lowering medication (vs. no medication	.99	.66–1.52	.996	.73	.55-.97	.028*	.85	.56–1.30	.448
PAM total	1.00	.99–1.02	.964	1.00	.99–1.01	.652	1.00	.99–1.02	.887
SCSCA									
*Blood glucose testing*	.97	.88–1.07	.527	.98	.91–1.06	.605	.97	.87–1.07	.553
*Foot-care*	1.00	.93–1.08	.944	1.06	1.00–1.13	.048*	1.01	.93–1.10	.853
EQ-5D total	.93	.43–2.00	.845	.80	.38–1.72	.560	.47	.21–1.04	.060
ADDQoL total	.96	.78–1.12	.668	1.04	.90–1.20	.628	.80	.65-.99	.037*
PAID total	1.02	.97–1.08	.429	1.01	.97–1.05	.690	.96	.91–1.01	.087
BIPQ									
*Consequence*	1.04	.94–1.15	.489	.99	.93–1.06	.845	1.04	.94–1.15	.466
*Timeline*	1.01	.94–1.08	.816	.98	.93–1.03	.393	.99	.91–1.07	.799
*Identity*	.97	.88–1.08	.594	1.04	.97–1.12	.266	1.06	.96–1.18	.230
*Illness concern*	1.01	.94–1.08	.754	1.05	.99–1.11	.099	.95	.87–1.04	.269
Social support–Hardly any (ref)									
A little	2.10	.73–6.06	.160	.93	.34–2.49	.870	1.16	.40–3.39	.783
Moderate	1.72	.60–5.11	4.92	.95	.37–2.45	.905	1.18	.38–3.68	.772
A lot	2.04	.78–5.31	.145	.84	.23–3.10	.778	.94	.23–3.76	.924
Patients with personal treatment goals (vs. those without)	.65	.43–1.00	.048*	1.52	1.09–2.13	.016*	2.64	1.54–4.55	.001*
Preferred monitoring frequency in the coming year (<4 times (ref))									
4 times	-	-	-	1.21	.33–4.43	.731	2.29	1.00–5.24	.050
>4 times a year	-	-	-	.91	.65–1.26	.569	1.11	.71–1.73	.639
Referral to other healthcare providers (vs. no referral)	.94	.64–1.36	.719	-	-	-	1.06	.68–1.64	.787
Change in medication (vs. no change)	1.11	.70–1.76	.650	1.08	.70–1.65	.729	-	-	-

OR: Odds Ratio; PC: Primary Care; SC: Secondary care; BP: blood pressure

* Significant (p<0.05) difference between PC and SC

## Discussion

This representative nationwide Dutch study demonstrates that the planned healthcare utilization of individuals with Type 2 diabetes is associated with both disease and person related factors. Many disease related factors differed significantly between patients treated in general practices compared to those treated in hospital-based outpatient clinics, which is a direct result of the organization of diabetes care in the Netherlands. Of note, patients treated in the general practices set different goals compared to patients treated in hospital outpatient clinics. After the person-centered annual consultation, 40% of the more ‘complex’ patients in outpatient clinics arranged a monitoring frequency of less than four times a year, compared to 19.5% of the patients in primary care. We can only speculate about the reason for this finding. Treating patients according to their preference may reduce healthcare costs [23–25]. Interestingly, also whether a physician or a nurse led the consultation determined the choices that have been made. Patients who had a consultation with a physician had a four times higher odds of a monitoring frequency of at least four times a year, whereas on the other hand physicians were much less likely to change people’s medication. It is known that monitoring frequencies are mainly determined by the physician, and to a lesser extent by patient factors and disease severity [25]. Our consultation model may enhance the process of mutually considering the value of diabetes monitoring visits by making the arrangement more explicit.

The HbA1c level was associated with both the arranged monitoring frequency and whether or not blood glucose lowering medication was changed; the use of blood glucose lowering medication also influenced the preferred monitoring frequency and referrals. This finding underpins the impact of diabetes control on diabetes related resource consumption and is in line with the results of a previous study that concluded that the use of blood glucose lowering medication is the strongest predictor of the healthcare utilization in general practice [22]. In this respect it is important to note that in our study more than 80% of all patients stated they made a shared decision on both treatment goals and about treatment and care [9], because patients who actively participate in the medical encounter have improved medication adherence [26–29], which makes diabetes care more efficient [30]. However, not only disease related factors were independently associated with the intended healthcare use. Also, a patient’s age, diabetes related quality of life, the need of foot care and whether individuals with Type 2 diabetes set specified goals before the annual consultation or not determined the care path following the consultation. In our opinion this finding justifies the application of our model, although one could have expected that more person related factors would have determined the utilization of diabetes care services on group level [31]. This means that on an individual level more factors are likely to influence the intended healthcare use. On an aggregate level health policy makers and guideline developers can hardly categorize patients into for example a category that needs more or a category that needs less diabetes care.

The strength of this study is its representative large sample of patients with Type 2 diabetes, physicians and nurses [9]. Furthermore, to our knowledge this is the first study that assessed the planned healthcare use in a person-centered context after implementation of a consultation model that addresses many if not all factors that determine diabetes self-management and that incorporates shared decision as a central feature.

Some limitations should also be considered. All data are observational, we could not assess whether a merely protocolled annual ‘control’ visit would have led to other treatment targets, treatment plans and ultimately resource consumption. Only a cluster randomized controlled trial could answer this question. Given the design without no pre-test question, we could only provide associations instead of causation of factors with the intended monitoring frequency, referrals to other healthcare providers and medication change. However, we think the implementation of our consultation model is justified if we want to take the recommendations from EASD and ADA seriously and to integrate patient’s preferences, needs, values and self-management possibilities into care.

Further research is recommended to assess the change of for example glycemic control, patient’s activation, illness perceptions, quality of life, distress one year after the use of our consultation model.

In conclusion, at the individual level this study shows that also person related factors affecting the preferred diabetes care use. If individuals with Type 2 diabetes are encouraged to set specified treatment goals and offered the opportunity to integrate their health-related quality of life into the diabetes care they prefer; and if these goals and preferences are part of a shared decision making process, it will result in individualized and person-centered healthcare utilization. Such an approach is a ‘real life’ possibility. At health care level, this study helps health policy makers identifying more suitable diabetes care.
